# Interaction between Notch signaling pathway and bioactive compounds and its intervention on cancer

**DOI:** 10.3389/fnut.2025.1647661

**Published:** 2025-09-23

**Authors:** Rong-Zu Nie, Hang Wang, Shuang-Shuang Wang, Huo-Min Luo, Chen Chen, Zhao-Hui Jing, Pei-Feng Li

**Affiliations:** ^1^College of Food and Bioengineering, Zhengzhou University of Light Industry, Zhengzhou, China; ^2^Institute of Life and Health, Zhengzhou University of Light Industry, Zhengzhou, China

**Keywords:** Notch signaling pathway, cancer, bioactive compounds, oncogenic role, cancer-suppressive role

## Abstract

The Notch signaling pathway is pivotal in cancer regulation, with its effects varying based on activation degree, tissue origin, and microenvironment. The dual role of Notch signaling is significant. It can promote or inhibit cancer progression depending on the context. This duality emphasizes the importance of nuanced therapeutic approaches. Recent research highlights natural bioactive compounds as modulators of Notch signaling, providing innovative insights for cancer prevention and therapy. This review explores the structural and functional mechanisms of Notch signaling in carcinogenesis and examines how natural compounds influence its activity, offering a foundation for targeted treatments. Bioactive compounds, such as flavonoids, non-flavonoids polyphenols, and terpenoids, show potential in modulating Notch signaling with low toxicity and multi-target effects. Compounds like resveratrol, curcumin, and EGCG inhibit key nodes in Notch signaling, reducing cancer cell proliferation and inflammation. Despite its promise, targeting Notch signaling poses challenges due to its complexity and variability across different cancers. Future research should focus on understanding the tissue-specific effects of Notch signaling, optimizing bioactive compound structures, and integrating basic and clinical studies to develop precision therapies. This review underscores the intricate role of Notch signaling in cancer and the transformative potential of bioactive compounds in therapeutic interventions.

## 1 Introduction

The Notch signaling pathway is a highly conserved intercellular communication mechanism found in both vertebrates and invertebrates, playing a critical role in embryonic development, cell differentiation, proliferation, apoptosis, and fate determination (1). The Notch signaling pathway consists of the Notch receptor, ligands, *γ*-secretase complex, and a series of downstream effector molecules (2). Its signaling is initiated through direct cell-to-cell contact, where ligand-receptor interactions trigger signal transmission (3). During this process, the Notch receptor undergoes two cleavages, releasing the intracellular domain (NICD), which translocates to the nucleus to regulate the expression of specific genes (4, 5). The precise regulation of Notch signaling is essential for maintaining normal tissue and organ function, and its aberrant activation or inhibition is closely associated with the onset of various diseases.

Recent studies indicate that the Notch signaling pathway plays a significant role in carcinogenesis (6). The mechanisms of Notch signaling in different tissues and cell types are diverse and complex, making it a potential pathogenic factor in certain conditions while serving a protective role in others. For example, in cancer, abnormal activation or inhibition of Notch signaling can promote cancer cell proliferation and metastasis, inhibit cell differentiation, and affect the cancer microenvironment (7). In certain cancers, such as acute lymphoblastic leukemia and breast cancer, excessive activation of Notch signaling leads to malignant cancer progression, whereas in other types of cancer, Notch signaling helps suppress carcinogenesis (8). Thus, Notch signaling plays a dual role in the pathogenesis and progression of cancer.

With the advancement of biochemical and molecular biology techniques, researchers have begun to focus on the interaction between Notch signaling and natural bioactive compounds. Bioactive compounds are substances extracted from natural resources such as plants, animals, and fungi that exhibit physiological activity. Common types include flavonoids, non-flavonoid polyphenols, terpenoids and other bioactive compounds (9). These compounds exhibit various bioactivities, including antioxidant, anti-inflammatory, anticancer, and immune-modulating properties. They are widely used in the prevention and treatment of chronic diseases like inflammatory disorders and cancer due to their low toxicity and multi-target effects (10). Compared to synthetic drugs, bioactive compounds have advantages such as natural origin, structural diversity, and fewer side effects, making them promising candidates in medicine, nutrition, and healthcare.

Studies have found that various natural compounds from plants can regulate Notch signaling through different mechanisms. For instance, some flavonoids inhibit *γ*-secretase activity, reducing the release of NICD, thereby blocking Notch activation; polyphenolic compounds like green tea polyphenols and resveratrol affect the expression of Notch target genes, preventing cancer cell proliferation and survival (11); terpenoids regulate other key nodes in the Notch signaling process, exerting multiple interventions in cancer (12). Moreover, different types of bioactive compounds exhibit synergistic effects in various disease models, offering new perspectives for combination therapies targeting multiple pathways.

The dual role of Notch signaling in the prevention and treatment of cancer offers both opportunities and challenges. Since Notch signaling produces markedly different effects under various physiological conditions, and its specific effects in different cell types and tissues vary, great caution is required when designing Notch-targeted intervention strategies. The structural diversity and relative safety of bioactive compounds provide a rich chemical library for modulating Notch signaling. With the deepening research into molecular-targeted therapies, these natural compounds can be chemically modified or integrated with nanotechnology to precisely target specific nodes in Notch signaling, enhancing their efficacy and specificity. In summary, the Notch signaling pathway plays a complex and variable role in cancer, and bioactive compounds demonstrate considerable potential in modulating this pathway.

## 2 Overview of the Notch signaling pathway headings

The Notch signaling pathway is a highly conserved intercellular communication mechanism that is widely involved in various physiological processes, such as embryonic development, tissue differentiation, cell proliferation, and apoptosis (13). In multicellular organisms, this pathway plays a crucial role in maintaining tissue homeostasis and coordinating cell behavior through the regulation of cell fate determination, tissue regeneration, and immune responses. The Notch signaling pathway consists of the Notch receptor, ligands, the *γ*-secretase complex, and a series of downstream transcription factors and target genes (14). Although its structure and signaling mechanism appear simple, the pathway is functionally flexible and complex.

### 2.1 Components of the Notch signaling pathway

The core components of the Notch signaling pathway include the Notch receptor, ligands, the *γ*-secretase complex, and downstream effector molecules. In mammals, there are four main Notch receptor subtypes: Notch1, Notch2, Notch3, and Notch4. The ligands include Delta-like ligands (DLL1, DLL3, DLL4) and Jagged family ligands (Jagged1 and Jagged2) (15). These ligands and receptors are single-pass transmembrane proteins that are widely distributed on the cell membrane. Activation of Notch signaling relies on direct cell-to-cell contact, playing a crucial role in the precise communication between cells in local tissue environments.

### 2.2 Activation mechanism of the Notch signaling pathway

**Figure 1:**
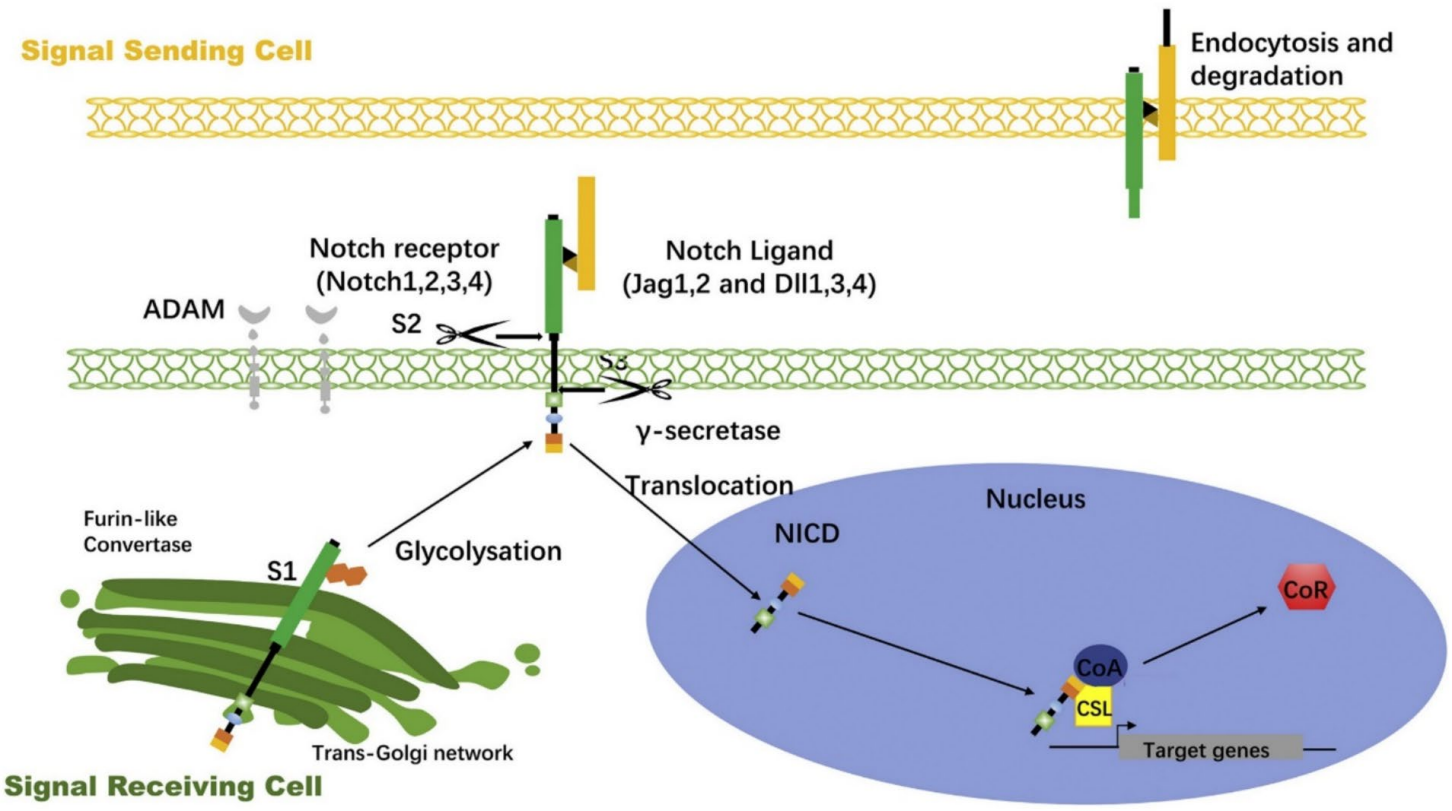
Notch signaling activation process (70).

The activation of Notch signaling begins with the binding of the ligand to the receptor (Figure 1). This typically occurs between two adjacent cells, where the ligand-expressing signaling cell interacts with the receptor-expressing responding cell. Upon ligand binding, the Notch receptor undergoes two cleavages (16). The first cleavage is mediated by metalloproteinases (ADAM family), which releases the extracellular portion of the receptor in a process known as S2 cleavage. Subsequently, the *γ*-secretase complex performs a second cleavage at the S3 site, producing the Notch intracellular domain (NICD) (17).

The NICD is the core active fragment of the Notch signaling pathway, possessing a transmembrane structure. After both cleavages, the NICD fragment enters the cytoplasm and rapidly translocates to the nucleus. In the nucleus, NICD binds to the transcription factor CSL (CBF1/RBP-Jκ/Suppressor of Hairless/LAG-1), forming a complex, and together with co-activators (e.g., MAML), it activates the transcription of target genes (18). Notch signaling target genes include Hes and Hey family genes, which play regulatory roles in cell fate determination and differentiation (19). NICD binds to the transcription factor and activates specific target genes, influencing cell division, differentiation, and apoptosis.

### 2.3 Negative feedback and regulatory mechanisms in Notch signaling

The activation of Notch signaling is tightly regulated by several mechanisms to ensure its spatial and temporal precision. First, the stability of NICD within the cell is strictly controlled. After NICD enters the nucleus and activates target genes, its stability gradually decreases, and it is eventually degraded through the ubiquitin-proteasome pathway. This degradation mechanism is essential for maintaining the transient and reversible nature of Notch signaling (20). Additionally, the regulation of Notch signaling involves several post-translational modifications, such as phosphorylation, acetylation, and glycosylation, which modulate the binding efficiency between Notch receptors and ligands, the sensitivity of receptor cleavage, and the nuclear translocation efficiency of NICD.

In the process of Notch signaling transmission during cell–cell contact, some cell surface factors, such as Fringe family proteins, can regulate the activity of Notch signaling by altering the ligand-receptor binding properties (21). For instance, Fringe modifies ligand binding through glycosylation, selectively enhancing or inhibiting specific Notch signal subtypes (22). Moreover, in the self-regulation of Notch signaling, some target genes (e.g., Hes1) inhibit Notch signaling through negative feedback mechanisms, forming a self-regulating loop that allows Notch signaling to adapt to changes in the cellular environment.

### 2.4 Physiological functions and diversity of Notch signaling

One of the notable characteristics of Notch signaling is its non-cell autonomous nature, meaning that the activation of Notch signaling in one cell can affect the behavior of adjacent cells (23). Therefore, Notch signaling is widely involved in the coordination between tissues and the allocation of cell fates. This pathway is especially important during embryonic development, where it regulates the development of the nervous, cardiovascular, and hematopoietic systems in vertebrates (24, 25). For example, Notch signaling plays a critical role in the differentiation of neural progenitor cells and stem cells, inhibiting their differentiation into specific lineages and maintaining their stem cell properties. Additionally, during the homeostasis and regeneration of adult tissues, Notch signaling regulates processes such as intestinal epithelial cell renewal, hematopoietic cell differentiation in the bone marrow, and angiogenesis (26). Under pathological conditions, abnormal activation or inhibition of Notch signaling is associated with various diseases. Overactive Notch signaling has been found to be closely linked with several types of cancer, such as acute T-cell leukemia, breast cancer, and hepatocellular carcinoma (27).

## 3 The role of Notch signaling in cancer

The role of the Notch signaling pathway in cancer is highly complex and exhibits duality. It can act as an oncogenic factor in some cancer types, while potentially functioning as a cancer suppressor in others (28, 29). This dual role depends on factors such as the tissue origin of the cancer, the microenvironment, and the extent of Notch signaling activation or inhibition (30). In certain cancers, Notch signaling promotes cancer progression and metastasis through mechanisms such as enhancing cell proliferation, inhibiting cell differentiation, and conferring resistance to apoptosis. However, in other cancer types, Notch signaling may act as a cancer suppressor by maintaining cell differentiation or preventing aberrant proliferation. A large body of research has confirmed that aberrant activation or inhibition of Notch signaling is closely associated with the initiation, progression, and metastasis of various cancers, including breast cancer, colon cancer, pancreatic cancer, and leukemia (27).

### 3.1 Oncogenic role of Notch signaling

In many types of cancer, the Notch signaling pathway is considered an oncogenic factor because it regulates cancer cell proliferation, survival, and invasiveness, thus promoting cancer initiation and progression (31). For example, in breast cancer, abnormal activation of Notch1 and Notch4 is closely associated with cancer formation and increased invasiveness. Studies have shown that Notch1 signaling can induce epithelial-mesenchymal transition (EMT) in breast cancer cells, enhancing their migratory capacity and promoting metastasis (32, 33). Activation of Notch signaling also inhibits cell differentiation, keeping cancer cells in an immature, proliferative state, a feature often linked to cancer stem cell properties. In breast cancer, the cancer stem cell populations activated by Notch1 and Notch4 signaling exhibit resistance to conventional chemotherapy and radiotherapy, leading to treatment failure and increased risk of relapse (34).

**Figure 2:**
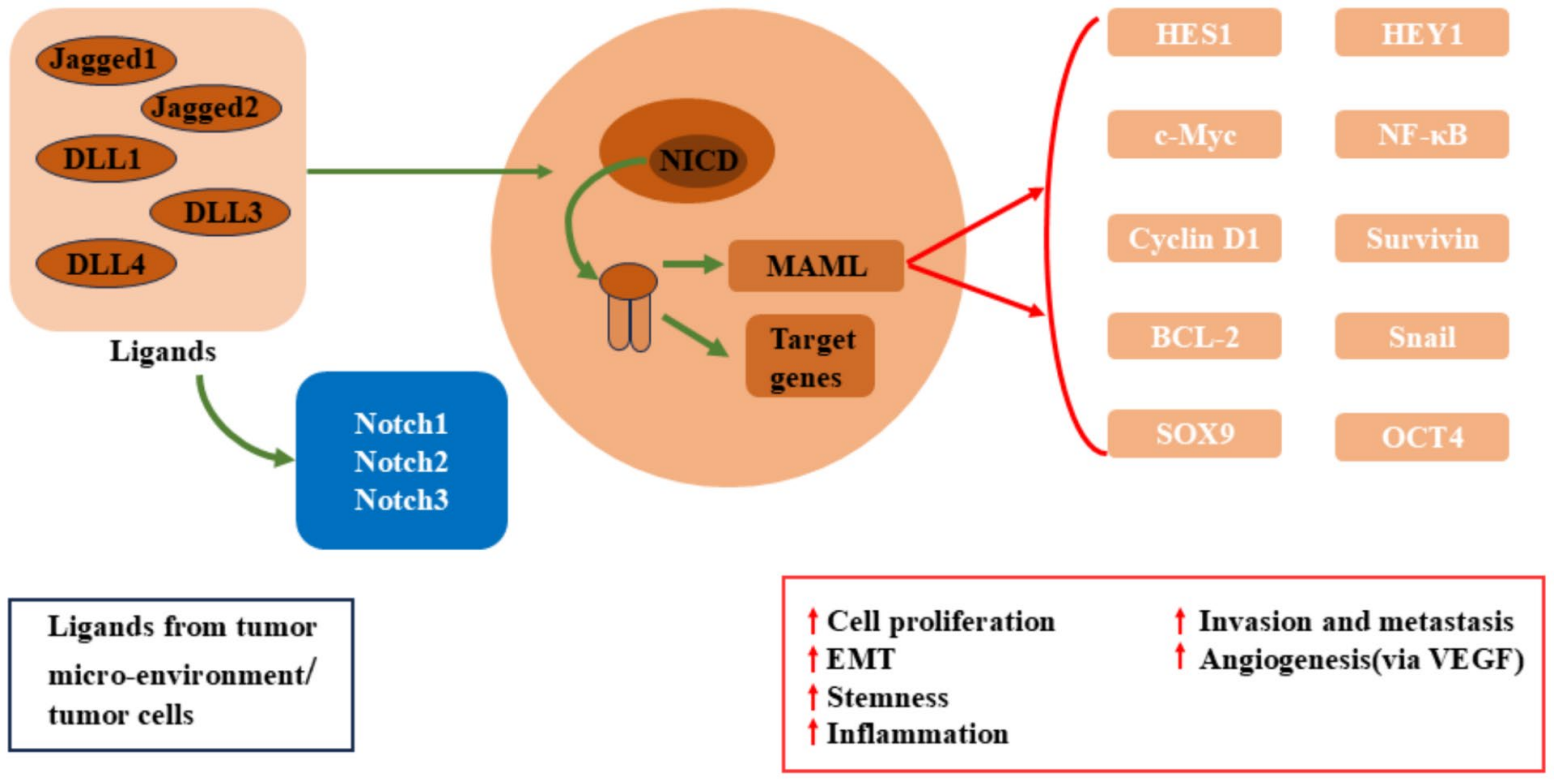
Dysregulated Notch signaling in breast cancer, colon cancer, pancreatic cancer.

The oncogenic role of Notch signaling has also been widely studied in colon cancer (Figure 2). In colon cancer, persistent activation of Notch signaling is typically accompanied by abnormal activity of the Wnt signaling pathway, with both pathways interacting to promote cancer cell proliferation and survival (35). In normal intestinal cells, Notch signaling maintains stem cell differentiation balance, ensuring the homeostatic renewal of the intestinal epithelium (36). However, excessive activation of Notch signaling leads to overproliferation and accumulation of precancerous cells, eventually leading to colon cancer formation. Additionally, Notch signaling enhances resistance to apoptosis, allowing cancer cells to survive in the harsh cancer microenvironment, further promoting cancer progression (37).

In pancreatic cancer, the cooperative interaction between Notch signaling and the oncogenic driver gene KRAS has been widely reported. KRAS mutations are common drivers of pancreatic cancer, and its upregulation activates Notch signaling, enhancing the proliferative capacity and drug resistance of pancreatic cancer cells (38). Overexpression of Notch1 and Notch3 in pancreatic cancer cells is closely associated with increased cancer invasiveness (39). Furthermore, Notch signaling plays a critical role in the cancer microenvironment by activating cancer-associated fibroblasts (CAFs) and promoting angiogenesis, helping cancer cells evade immune surveillance and acquire more nutrients, thus accelerating pancreatic cancer progression (40).

### 3.2 Cancer-suppressive role of Notch signaling

Although Notch signaling exhibits oncogenic effects in many cancers, it also has cancer-suppressive roles in certain cancer types. Specifically, activation of Notch signaling can inhibit cancer cell proliferation or induce differentiation, thus suppressing cancer progression. A classic example is the inactivation of Notch1 mutations, which are often closely associated with carcinogenesis in these cancer types. In normal skin tissue, Notch1 primarily promotes the differentiation of keratinocytes, and its suppressive role helps maintain normal tissue structure (41). However, when Notch1 signaling is inactivated, uncontrolled keratinocyte proliferation occurs, leading to carcinogenesis (42). Studies have shown that reactivating Notch1 signaling can effectively suppress the proliferation of these cancer cells and partially restore their differentiation ability.

Similarly, the role of Notch signaling in small cell lung cancer (SCLC) reflects its cancer-suppressive effect. Small cell lung cancer is a highly aggressive and malignant form of lung cancer, and Notch signaling is typically low in these cancers (43). The low expression of Notch1 and Notch2 is closely associated with uncontrolled cancer proliferation, suggesting that Notch signaling plays a potential cancer-suppressive role in maintaining normal cell growth control. By activating Notch signaling, the proliferation of small cell lung cancer cells can be suppressed, and their sensitivity to chemotherapy drugs is enhanced (44). Thus, in this cancer type, Notch signaling may suppress cancer growth by maintaining normal cell cycle regulation.

### 3.3 The role of Notch signaling in the cancer microenvironment

Notch signaling not only directly affects cancer cell proliferation and differentiation but also plays multiple roles in the cancer microenvironment. The cancer microenvironment includes fibroblasts, immune cells, endothelial cells, and extracellular matrix components, all of which interact to influence cancer growth and metastasis (45). Notch signaling regulates the activity of cancer-associated fibroblasts (CAFs) and promotes extracellular matrix remodeling, thus providing a more suitable environment for cancer cell growth. CAFs secrete pro-inflammatory and pro-angiogenic factors in the cancer microenvironment, and activation of Notch signaling enhances these processes, facilitating cancer angiogenesis and cancer cell invasion (46).

Moreover, Notch signaling also regulates the distribution and activity of immune cells in the cancer microenvironment, influencing cancer progression. Studies have shown that Notch signaling can help cancer cells evade immune surveillance by suppressing anti-cancer immune responses. For example, activation of Notch signaling plays an important role in macrophage polarization within the cancer microenvironment. By promoting macrophage polarization to the M2 phenotype, Notch signaling suppresses anti-cancer immune responses and enhances cancer invasiveness (47). Additionally, Notch signaling is closely linked to T cell differentiation, where it regulates the ratio of Treg cells and Th17 cells, thus modulating immune tolerance and pro-inflammatory responses within the cancer microenvironment.

### 3.4 Notch signaling as a potential target in cancer therapy

The dual role of Notch signaling makes it a potential therapeutic target in cancer treatment, but its complexity also increases the difficulty of targeted regulation. Inhibitors and activators of Notch signaling have shown therapeutic potential in various cancer types. *γ*-Secretase inhibitors (GSIs), which block the generation of NICD, are the most studied Notch signaling inhibitors (48). In cancers with high Notch signaling activity, such as breast cancer and pancreatic cancer, GSIs have shown effects in inhibiting cancer growth (49). However, because *γ*-secretase also plays a role in other signaling pathways, the use of GSIs may lead to adverse effects, presenting challenges for their clinical application.

Recently, the specific targeted regulation of Notch signaling has become a new research focus. For example, antibody-based Notch receptor blockers can selectively inhibit specific Notch receptor subtypes, thereby reducing side effects. Moreover, targeting specific upstream or downstream elements of the Notch signaling pathway, such as the nuclear translocation of NICD or specific transcriptional target genes, is also considered an effective intervention approach.

## 4 Regulation of Notch signaling by bioactive compounds

In recent years, natural bioactive compounds have gained attention as interventions due to their broad pharmacological effects and relatively low toxicity. Particularly in the field of anticancer therapy, bioactive compounds have been increasingly recognized for their ability to regulate the Notch signaling pathway. Research has shown that many natural compounds can interfere with different stages of Notch signaling, inhibiting its pro-pathological effects in cancer, thus achieving therapeutic outcomes (9). The following are some common bioactive compounds and their specific mechanisms of action in modulating Notch signaling.

### 4.1 Flavonoids

**Figure 3:**
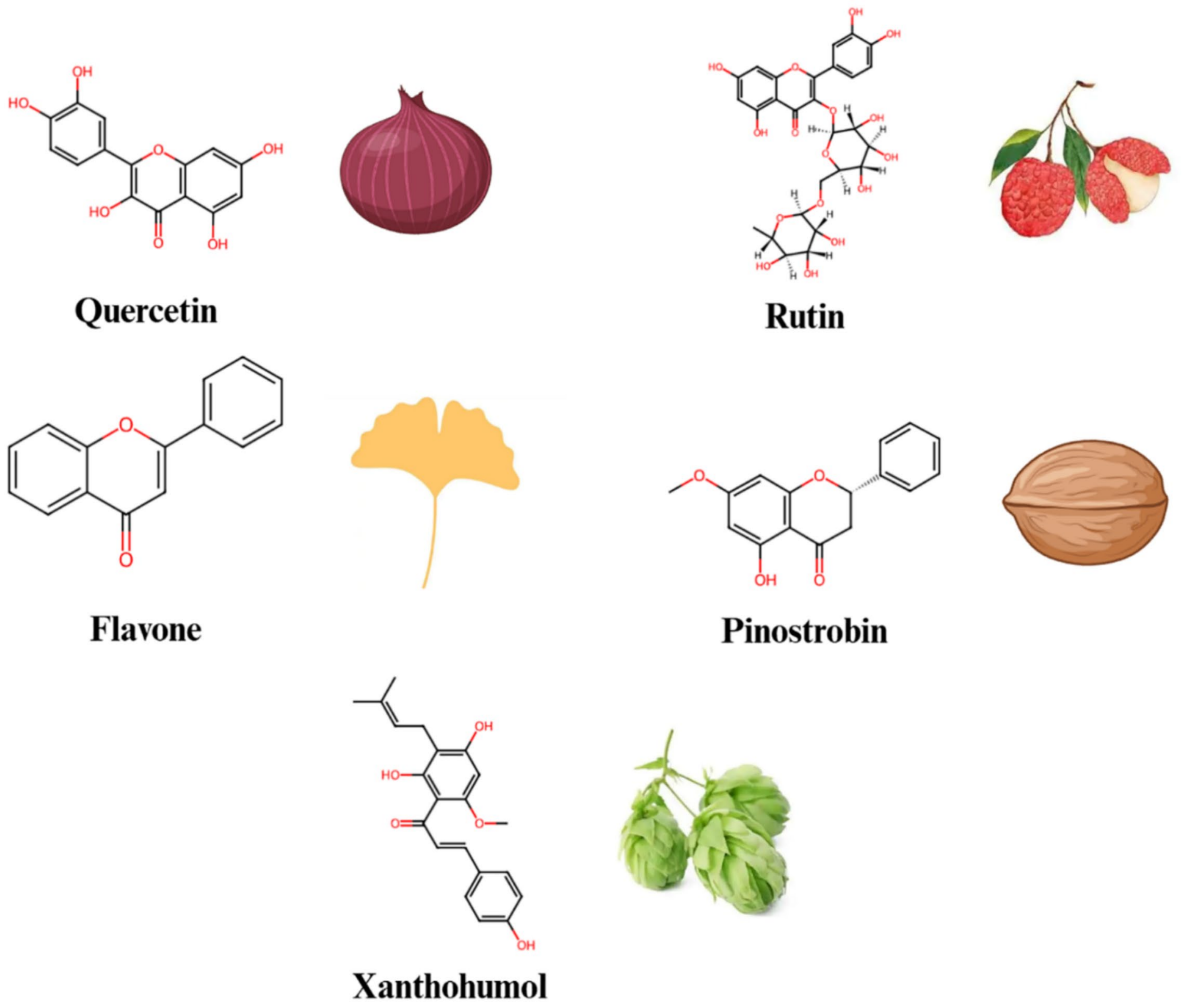
The chemical structures and sources of flavonoid compounds that can interact with the Notch signaling pathway.

Flavonoids (Figure 3) are a group of polyphenolic compounds widely found in plants, known for their antioxidant, anti-inflammatory, and anticancer activities (Table 1). Quercetin is one of the widely studied flavonoids (50). Quercetin has been demonstrated to regulate Notch signaling (51). Research shows that quercetin can inhibit *γ*-secretase activity, reducing the production of NICD and downregulating Notch target gene expression (52). In inflammatory environments, quercetin effectively reduces the expression of pro-inflammatory cytokines such as IL-6 and TNF-*α*, alleviating inflammation (53). Additionally, quercetin induces apoptosis in cancer cells by reducing Notch signaling activation.

**Table 1:** The effects of different polyphenolic compounds on the Notch signaling pathway and their molecular mechanisms.

Name of natural food factor	Function	Molecular mechanism
Quercetin	Antioxidant, anti-inflammatory, anti-cancer	Inhibits γ-secretase activity, reduces NICD production, and downregulates Notch target gene expression
Total flavonoids of *litchi* seed	Inhibiting breast cancer and eliminating breast cancer stem cells	Inhibiting breast cancer and eliminating breast cancer stem cells by blocking the Notch3 signaling pathway
Flavone	Inhibits T-ALL cell proliferation	Downregulation of Notch1 signaling pathway induces ICN1 ubiquitination and degradation via c-Cbl
Pinostrobin	A549 cell apoptosis	Downregulation of Notch1, Jagged-1, and Hes-1 expression
Xanthohumol	Inhibit pancreatic cancer cell growth	Enhanced apoptosis by inhibiting the Notch1 signaling pathway
Epigallocatechin gallate (EGCG)	Antioxidant, anti-inflammatory, anti-cancer	Reduce NICD production, downregulate Notch target gene expression, and inhibit cancer cell proliferation, migration, and invasion
Caffeic acid	Anti-inflammatory, anti-cancer	Inhibit Notch signaling activity and block cancer cell proliferation signals
Resveratrol	Anti-inflammatory, anti-cancer	Downregulates Notch receptor and ligand expression, reduces NICD production, and inhibits Notch signaling pathway
Ellagic acid	Anti-inflammatory, anti-cancer, antioxidant	Inhibition of Akt and Notch signaling

*Litchi chinensis* Sonn (Litchi) belongs to the Sapindaceae family. For decades, litchi seeds and litchi seed formulas have been used clinically in China to prevent and treat breast cancer, as well as to enhance the quality of life for breast cancer patients (54, 55). Liao et al. (56) examined the effects and mechanisms of litchi seed total flavonoids in both *in vitro* and *in vivo* studies. They found that the total flavonoids from litchi seed could inhibit the breast cancer and eliminate breast cancer stem cells by blocking the Notch3 signaling pathway. Among the total flavonoids of litchi seed, rutin might be responsible for the suppressive effects of total flavonoids of litchi seed on breast cancer stem cells.

Zhu et al. (57) revealed that the flavone inhibited cell proliferation by down-regulating the Notch1 signaling pathway in CCRF-CEM and Molt-4 T-ALL (T-cell acute lymphoblastic leukemia) cells. The increase in c-Cbl level induced by the flavone enhanced its interactions with ICN1 (Intracellular Notch1), leading to the ubiquitinylation and degradation of ICN1. Knockdown of c-Cbl reversed flavone-induced down-regulating of ICN1 and the inhibition of cell proliferation in T-ALL cells. Consequently, there findings provided experimental support for the development of flavone as a potential leukemia treatment and c-Cbl as a new target for anti-Notch1 therapy in T-ALL.

Pinostrobin (PN) is a dietary bioflavonoid naturally present in various medicinal plants (58). PN triggered the generation of reactive oxygen species (ROS) in A549 cells, which activated key apoptosis regulators such as Bax, Bad and Bcl2, as well as lead to the release of cytochrome c, and the activation of the caspase cascade, including caspase-9 and -3. Furthermore, PN treatment of A549 cells resulted in a dose-dependent decrease in the expression levels of Notch1, Jagged-1 and Hes-1 (59). These results demonstrated that PN inhibited the growth of A549 cells by down-regulating the Notch pathway.

Xanthohumol is a natural flavonoid derived from the cones of hops (*Humulus lupulus* Linn.) (60). Kunnimalaiyaan et al. (61) evaluated the effectiveness of xanthohumol on pancreatic cancer cell lines (AsPC-1, PANC-1, L3.6pl, MiaPaCa-2, 512, and 651) in terms of inhibiting cell growth, using real-time and colony-forming assays. Treatment with xanthohumol resulted in a decrease in cellular proliferation in a dose- and time-dependent manner. The growth-inhibitory effect of xanthohumol on pancreatic cancer cell lines is attributed to enhanced apoptosis through the inhibition of the Notch1 signaling pathway, as demonstrated by the reduction of Notch1, Hes-1, and survivin at both mRNA and protein levels. These findings clearly indicated that the growth-inhibitory effect of xanthohumol in pancreatic cancer cells is primarily mediated by the reduction of Notch1.

### 4.2 Non-flavonoid polyphenolic compounds

**Figure 4:**
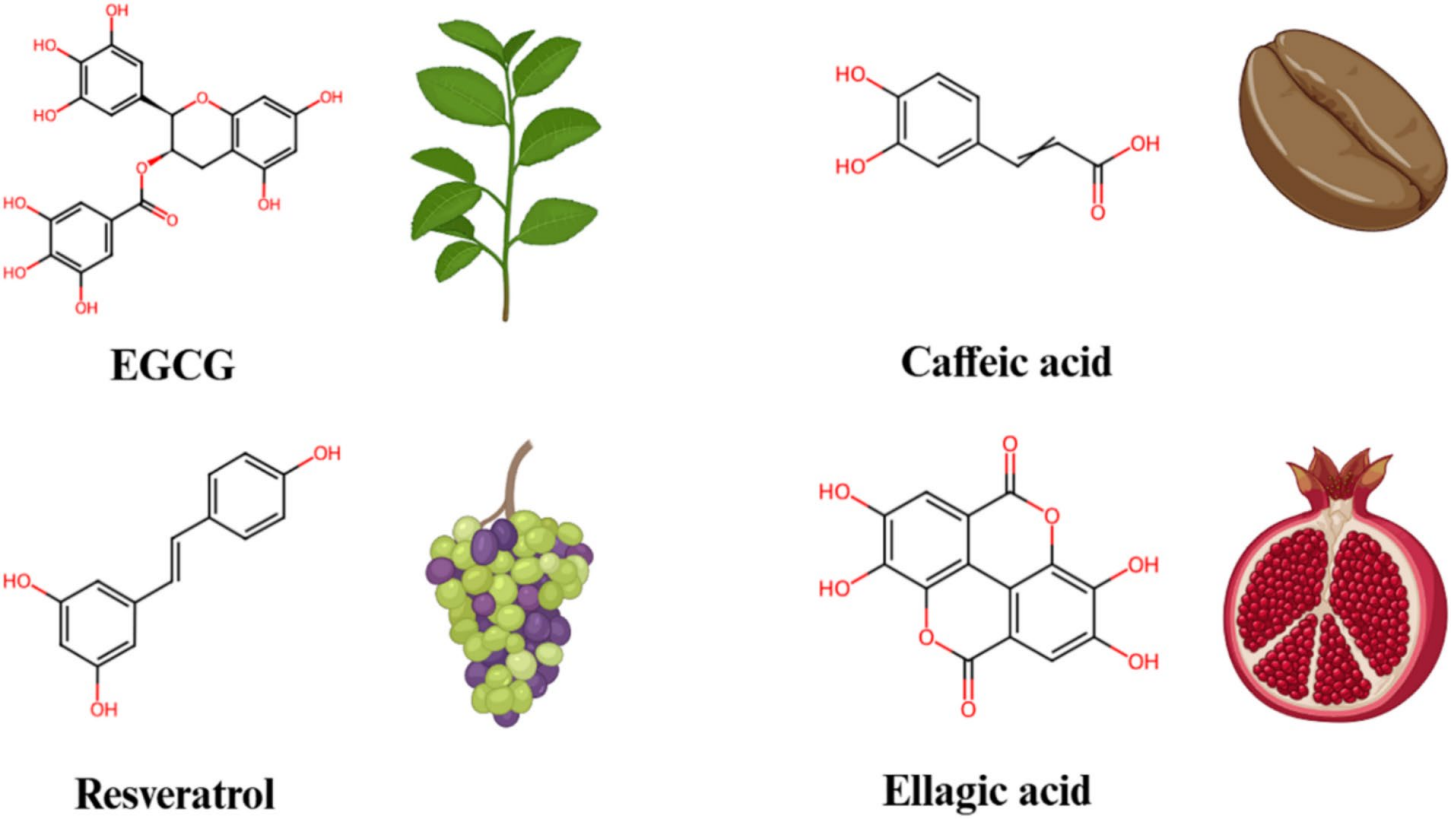
The chemical structures and sources of non-flavonoid polyphenols that can interact with the Notch signaling pathway.

Non-flavonoids polyphenolic compounds (Figure 4) are widely found in plants and have various pharmacological effects, including antioxidant, anti-inflammatory, and anticancer properties. Green tea polyphenols and caffeic acid are typical representatives of this class of compounds. Epigallocatechin gallate (EGCG), the main component in green tea polyphenols, has been shown to significantly inhibit Notch signaling (62). Research shows that EGCG regulates Notch target gene expression by reducing NICD production, thereby inhibiting cancer cell proliferation, migration, and invasiveness (63). EGCG’s anticancer effects have been widely reported in models of breast cancer, lung cancer, and others (64). Both Notch1 and Notch2 were observed to be overexpressed in tongue squamous cell carcinoma in comparison to their levels in normal tongue cells. As a potent agent, EGCG inhibited the activation of the Notch signaling pathway, leading to significant deregulation of downstream target genes such as Hey1, CyclinD1 and CDK4, which in turn modulated the expression level of Notch1 and enhanced the sensitivity of tongue cancer cells to EGCG (65). Additionally, EGCG can reduce the production of pro-inflammatory cytokines, alleviating inflammation and demonstrating significant anti-inflammatory effects in some inflammatory disease models.

Caffeic acid, a common plant polyphenol found in coffee, grains, and fruits, also exhibits significant anti-inflammatory and anticancer effects. Studies have found that caffeic acid inhibits Notch signaling activity and blocks the proliferation signaling of cancer cells (66). In certain cancer cell models, caffeic acid induces apoptosis by inhibiting Notch signaling. Moreover, caffeic acid reduces the expression of pro-inflammatory cytokines, alleviating inflammation, and shows potential in chronic inflammatory diseases.

Resveratrol, found in grape skins and red wine, has been demonstrated to significantly inhibit Notch signaling. Specifically, resveratrol downregulates the expression of Notch receptors and ligands, which leads to a decrease in the production of NICD, thereby inhibiting the activation of Notch signaling pathway (67). Previous study indicated that, in neuroendocrine cancers, administration of resveratrol at doses of 2.5 g/twice a day suppressed the cancer growth and invasion by modulating the Notch-1 signaling pathway (68). Additionally, research has also shown that this regulatory effect of resveratrol can decrease the secretion of pro-inflammatory factors, thus alleviating inflammation in models of inflammatory disease (69). Furthermore, resveratrol can inhibit the proliferation of cancer cells by activating apoptosis pathways, particularly in models of breast cancer, colon cancer, and others, demonstrating significant anticancer effects.

Ellagic acid, a dimeric derivative of gallic acid, is present in a variety of fruits, vegetables, and wines, such as persimmon, pomegranates, raspberries, black raspberries, strawberries (70). Ellagic acid is widely recognized for its potent anti-inflammatory, anticancer, antioxidant and antidiabetic properties (71, 72). Wang et al. (73) investigated the effects of ellagic acid on cancer growth in glioblastoma xenografted mice. They found that treatment with ellagic acid significantly suppressed cancer growth in these xenografted models. Furthermore, a marked reduction in Akt and Notch signaling was observed in the xenografts of cancer-bearing mice treated with ellagic acid. These findings clearly suggested that ellagic acid might be advantageous in the treatment of glioblastoma.

### 4.3 Terpenoids

Terpenoids are widely found in plants and exhibit various bioactivities, including anti-cancer, anti-inflammatory, and antimicrobial effects (Table 2) (74). Among them, curcumin, ursolic acid and carvacrol are well-known terpenoids that have been extensively studied. Curcumin, a natural compound extracted from turmeric, has significant anti-inflammatory and anticancer properties (75). Studies have shown that curcumin exerts its anticancer effects by inhibiting Notch signaling activity (76). Specifically, curcumin interferes with Notch receptor activation and the generation of NICD, thereby inhibiting Notch signaling and suppressing cancer cell proliferation and survival, especially in models of colon cancer, breast cancer, and others, where it shows good anticancer effects (77, 78). At the same time, curcumin also reduces the production of pro-inflammatory factors, alleviating inflammation, and demonstrates significant anti-inflammatory effects in inflammatory disease models (79).

**Table 2:** The effects of bioactive compounds other than polyphenols on the Notch signaling pathway and their molecular mechanisms.

Name of natural food factor	Function	Molecular mechanism
Curcumin	Anti-inflammatory, anti-cancer	Inhibit Notch signaling activity, reduce NICD production, and inhibit cancer cell proliferation and survival
Ursolic acid	Anti-cancer, anti-inflammatory	Inhibits γ-secretase activity, reduces NICD production, inhibits cancer cell proliferation and induces apoptosis
Carvacrol	Antioxidant, anti-inflammatory, anti-cancer	Downregulation of Notch-1 and Jagged-1 expression
Diallyl trisulfide	Anti-cancer, anti-inflammatory	Reduce Notch receptor and ligand expression, inhibit Notch signaling pathway
Berberine	Anti-inflammatory, anti-cancer	Interfere with Notch signaling activity and reduce the secretion of pro-inflammatory factors

Ursolic acid, a natural triterpenoid compound found in many plants, has been shown to have both anticancer and anti-inflammatory effects. Ursolic acid inhibits *γ*-secretase activity, thereby reducing the production of NICD and suppressing Notch signaling activation (80). Research indicates that ursolic acid significantly inhibits cancer cell proliferation and induces apoptosis, particularly in cancer models of lung and liver cancer. Additionally, ursolic acid has notable anti-inflammatory properties. By regulating Notch signaling, it reduces the expression of pro-inflammatory cytokines, providing protective effects in inflammatory diseases (81).

Carvacrol is a monoterpenoid phenol predominantly found in the essential oils of plants from the Lamiaceae family. Numerous previous studies have highlighted various biological activities of carvacrol, including its antioxidant, anti-inflammatory and anticancer properties. Khan et al. (82) investigated the chemopreventive and therapeutic effects of carvacrol against the prostate cancer cell line PC-3, along with its detailed mechanism of action. They discovered that treatment with carvacrol significantly decreased the viability of PC-3 cells in a dose- and time-dependent manner. Additionally, they demonstrated that carvacrol could inhibit Notch signaling in PC-3 cells by downregulating Notch-1, and Jagged-1.

### 4.4 Other bioactive compounds

In addition to the above-mentioned bioactive compounds, many other natural products have been found to regulate Notch signaling. Sulfur compounds and alkaloids are examples of such compounds. For instance, diallyl trisulfide (DATS) from garlic, a sulfur compound, inhibits the activation of Notch signaling by decreasing the expression levels of Notch receptors and ligands. As a result, DATS demonstrates significant anticancer effects in *in vitro* cancer models (83). Moreover, DATS also reduces the production of pro-inflammatory cytokines, offering protective effects in various inflammatory diseases.

Berberine, an alkaloid widely found in the plant *Coptis chinensis*, has both anti-inflammatory and anticancer properties (84). Studies show that berberine interferes with Notch signaling activity, reducing the secretion of pro-inflammatory factors and thus alleviating inflammation (10). Additionally, berberine inhibits the proliferation of various cancer cells and induces apoptosis, contributing to its anticancer effects. These effects of berberine have been demonstrated in multiple cancer models, including colon cancer and leukemia.

## 5 The potential and challenges of Notch signaling as a therapeutic target

Due to its important regulatory role in various pathological processes, particularly its multifaceted effects in cancer, the Notch signaling pathway holds great promise as a therapeutic target. However, the dual effects of Notch signaling in these diseases also present significant challenges. In different types of cancers, Notch signaling may have either a promoting or inhibiting effect. Therefore, designing targeted therapies based on Notch signaling requires careful selection of the appropriate modulation strategy to avoid or minimize side effects.

One key consideration in designing targeted therapies is the tissue-specific effects of Notch signaling. The functional differences of this pathway in various tissues and cell types mean that a single Notch inhibitor or agonist may not have a universal therapeutic effect across different diseases. For example, Notch1 signaling has an oncogenic effect in some cancers such as breast cancer but acts as a cancer suppressor in others, such as skin cancer (85). Therapeutic strategies need to consider these differential effects and analyze the specific role of particular Notch signaling components in various cancer to select the most suitable intervention.

Time dependency is another important factor to consider in Notch signaling-targeted therapy. The role of Notch signaling in key processes such as cell proliferation, differentiation, and apoptosis often depends on the precise timing of its regulation. Therefore, intervention strategies targeting the Notch pathway must precisely regulate the timing of signal activation to avoid potential negative effects. Developing modulators that can dynamically adjust Notch signaling activity, rather than merely inhibiting or activating it, will be crucial for achieving optimal therapeutic outcomes.

In addition to tissue specificity and time dependence, structural modification of bioactive compounds offers new opportunities for targeting Notch signaling in therapy. In recent years, structural modification and optimization of natural bioactive compounds have significantly improved their selectivity, efficacy, and pharmacokinetic properties. For example, modifying the molecular structure of flavonoids and polyphenolic compounds can enhance their binding efficiency to specific Notch family members while reducing interference with other signaling pathways (86). Through rational structural optimization, the efficacy of these compounds can be improved, and their specificity enhanced, thereby reducing side effects in non-target tissues. Furthermore, metabolic stability and bioavailability issues can also be addressed through structural modifications, improving their therapeutic effectiveness *in vivo*. In summary, although the Notch signaling pathway holds great potential as a therapeutic target, its complex dual effects and the challenges in applying bioactive compounds to target this pathway in therapy remain significant. Future research should focus on further understanding the specific regulatory mechanisms of Notch signaling in different pathological environments, optimizing the structure of bioactive compounds to enhance selectivity and stability, and facilitating the clinical application of Notch-targeted therapies.

## 6 Prospects and conclusion

The complex mechanisms of Notch signaling in cancer provide both rich possibilities and challenges for targeted modulation of this pathway. Although aberrant activation or inhibition of Notch signaling plays a key role in many disease processes, the tissue specificity and time dependence of this pathway mean that a one-size-fits-all approach to regulation is unlikely to be effective. Precision therapies targeting Notch not only need to understand the role of this pathway in specific diseases but also must consider individual patient differences and the unique features of each disease.

Bioactive compounds, with their natural low toxicity and diverse physiological activities, show broad potential in regulating Notch signaling. These compounds can selectively modulate Notch signaling and interact with other signaling pathways, offering multiple therapeutic effects in the treatment of cancer. Through in-depth studies of polyphenols, flavonoids, terpenoids, and other bioactive compounds, scientists have found that these natural molecules can influence multiple stages of Notch signaling to regulate cancer development. In specific cancer models, these compounds exhibit significant anticancer activity, while also showing pronounced anti-inflammatory effects in models of inflammatory diseases.

Future research should focus on several key areas: first, further elucidating the specific mechanisms of Notch signaling in different types of cancer to design more targeted treatment strategies; second, exploring structural optimization of bioactive compounds to improve their targeting specificity and *in vivo* stability, thereby enhancing their therapeutic efficacy and safety; and finally, emphasizing the integration of *in vitro* and in vivo studies to promote the translation of basic research into clinical applications, providing a solid foundation for the use of Notch-targeted therapies in treating cancer.

In conclusion, the multifunctional regulatory role of Notch signaling in cancer offers vast potential for therapeutic strategies targeting this pathway. Bioactive compounds, as potential modulators of this signaling, also demonstrate significant application potential. Future research should aim to integrate basic science with clinical trials, optimizing bioactive compound design and deepening our understanding of Notch signaling to gradually achieve precision therapies targeting Notch, offering new treatment options for patients with inflammation and cancer.

## References

[1] Mohr OL. Character changes caused by mutation of an entire region of a chromosome in *Drosophila*. Genetics. (1919) 4:275–82. doi: 10.1093/genetics/4.3.275, PMID: 17245926 PMC1200460

[2] Austin J Kimble J. Transcript analysis of glp-1 and lin-12, homologous genes required for cell interactions during development of *C. elegans*. Cell. (1989) 58:565–71. doi: 10.1016/0092-8674(89)90437-6, PMID: 2758467

[3] Yochem J Weston K Greenwald I. The *Caenorhabditis elegans* lin-12 gene encodes a transmembrane protein with overall similarity to *Drosophila* notch. Nature. (1988) 335:547–50. doi: 10.1038/335547a0, PMID: 3419531

[4] Tan H Xu W Ding X Ye H Hu Y He X. Notch/NICD/RBP-J signaling axis regulates M1 polarization of macrophages mediated by advanced glycation end products. Glycoconj J. (2022) 39:487–97. doi: 10.1007/s10719-022-10062-y, PMID: 35666407

[5] Wu J Bala Tannan N Vuong LT Koca Y Collu GM Mlodzik M. Par3/bazooka binds NICD and promotes notch signaling during *Drosophila* development. Dev Biol. (2024) 514:37–49. doi: 10.1016/j.ydbio.2024.06.009, PMID: 38885804 PMC11287782

[6] Shang Y Smith S Hu X. Role of notch signaling in regulating innate immunity and inflammation in health and disease. Protein Cell. (2016) 7:159–74. doi: 10.1007/s13238-016-0250-0, PMID: 26936847 PMC4791423

[7] Bolós V Blanco M Medina V Aparicio G Díaz-Prado S Grande E. Notch signalling in cancer stem cells. Clin Transl Oncol. (2009) 11:11–9. doi: 10.1007/s12094-009-0305-2, PMID: 19155199

[8] Meurette O Mehlen P. Notch signaling in the tumor microenvironment. Cancer Cell. (2018) 34:536–48. doi: 10.1016/j.ccell.2018.07.009, PMID: 30146333

[9] Cao L Yang Y Ye Z Lin B Zeng J Li C. Quercetin-3-methyl ether suppresses human breast cancer stem cell formation by inhibiting the Notch1 and PI3K/Akt signaling pathways. Int J Mol Med. (2018) 42:1625–36. doi: 10.3892/ijmm.2018.3741, PMID: 29956731

[10] Wang M Sun L Wang L Sun Y. Effects of Berberine on circular RNA expression profiles in human gastric Cancer cells. Evid Based Complement Alternat Med. (2021) 2021:1–16. doi: 10.1155/2021/6688629, PMID: 34055022 PMC8112944

[11] Cecchinato V Chiaramonte R Nizzardo M Cristofaro B Basile A Sherbet GV. Resveratrol-induced apoptosis in human T-cell acute lymphoblastic leukaemia MOLT-4 cells. Biochem Pharmacol. (2007) 74:1568–74. doi: 10.1016/j.bcp.2007.08.001, PMID: 17868649

[12] Chen J Chang H Peng X Gu Y Yi L Zhang Q. 3,6-dihydroxyflavone suppresses the epithelial-mesenchymal transition in breast cancer cells by inhibiting the notch signaling pathway. Sci Rep. (2016) 6:28858. doi: 10.1038/srep28858, PMID: 27345219 PMC4921838

[13] Zhou B Lin W Long Y Yang Y Zhang H Wu K. Notch signaling pathway: architecture, disease, and therapeutics. Signal Transduct Target Ther. (2022) 7:95. doi: 10.1038/s41392-022-00934-y, PMID: 35332121 PMC8948217

[14] Goruganthu MUL Shanker A Dikov MM Carbone DP. Specific targeting of notch ligand-receptor interactions to modulate immune responses: a review of clinical and preclinical findings. Front Immunol. (2020) 11:1958. doi: 10.3389/fimmu.2020.01958, PMID: 32922403 PMC7456812

[15] Rehman AO Wang C-Y. Notch signaling in the regulation of tumor angiogenesis. Trends Cell Biol. (2006) 16:293–300. doi: 10.1016/j.tcb.2006.04.003, PMID: 16697642

[16] Tamura K Taniguchi Y Minoguchi S Sakai T Tun T Furukawa T. Physical interaction between a novel domain of the receptor notch and the transcription factor RBP-Jκ/Su(H). Curr Biol. (1995) 5:1416–23. doi: 10.1016/S0960-9822(95)00279-X, PMID: 8749394

[17] De Strooper B Annaert W Cupers P Saftig P Craessaerts K Mumm JS. A presenilin-1-dependent γ-secretase-like protease mediates release of notch intracellular domain. Nature. (1999) 398:518–22. doi: 10.1038/19083, PMID: 10206645

[18] Hildebrand D Uhle F Sahin D Krauser U Weigand MA Heeg K. The interplay of notch signaling and STAT3 in TLR-activated human primary monocytes. Front Cell Infect Microbiol. (2018) 8:241. doi: 10.3389/fcimb.2018.00241, PMID: 30042932 PMC6048282

[19] Bray SJ. Notch signalling in context. Nat Rev Mol Cell Biol. (2016) 17:722–35. doi: 10.1038/nrm.2016.94, PMID: 27507209

[20] Pagie S Gérard N Charreau B. Notch signaling triggered via the ligand DLL4 impedes M2 macrophage differentiation and promotes their apoptosis. Cell Commun Signal. (2018) 16:4. doi: 10.1186/s12964-017-0214-x, PMID: 29321062 PMC5764024

[21] Nowell CS Radtke F. Notch as a tumour suppressor. Nat Rev Cancer. (2017) 17:145–59. doi: 10.1038/nrc.2016.145, PMID: 28154375

[22] Acar M Jafar-Nejad H Takeuchi H Rajan A Ibrani D Rana NA. Rumi is a CAP10 domain glycosyltransferase that modifies notch and is required for notch signaling. Cell. (2008) 132:247–58. doi: 10.1016/j.cell.2007.12.016, PMID: 18243100 PMC2275919

[23] Hilton MJ Tu X Wu X Bai S Zhao H Kobayashi T. Notch signaling maintains bone marrow mesenchymal progenitors by suppressing osteoblast differentiation. Nat Med. (2008) 14:306–14. doi: 10.1038/nm1716, PMID: 18297083 PMC2740725

[24] Alabi RO Farber G Blobel CP. Intriguing roles for endothelial ADAM10/notch signaling in the development of organ-specific vascular beds. Physiol Rev. (2018) 98:2025–61. doi: 10.1152/physrev.00029.2017, PMID: 30067156 PMC6442920

[25] Herbert SP Stainier DYR. Molecular control of endothelial cell behaviour during blood vessel morphogenesis. Nat Rev Mol Cell Biol. (2011) 12:551–64. doi: 10.1038/nrm3176, PMID: 21860391 PMC3319719

[26] Dong Y Jesse AM Kohn A Gunnell LM Honjo T Zuscik MJ. RBPjκ-dependent notch signaling regulates mesenchymal progenitor cell proliferation and differentiation during skeletal development. Development. (2010) 137:1461–71. doi: 10.1242/dev.042911, PMID: 20335360 PMC2853848

[27] Aster JC Pear WS Blacklow SC. The varied roles of notch in Cancer. Ann Rev Pathol. (2017) 12:245–75. doi: 10.1146/annurev-pathol-052016-100127 27959635 PMC5933931

[28] Reynolds TC Smith SD Sklar J. Analysis of DNA surrounding the breakpoints of chromosomal translocations involving the β T cell receptor gene in human lymphoblastic neoplasms. Cell. (1987) 50:107–17. doi: 10.1016/0092-8674(87)90667-2 3036364

[29] Ellisen LW Bird J West DC Soreng AL Reynolds TC Smith SD. TAN-1, the human homolog of the Drosophila notch gene, is broken by chromosomal translocations in T lymphoblastic neoplasms. Cell. (1991) 66:649–61. doi: 10.1016/0092-8674(91)90111-B, PMID: 1831692

[30] Wang S Gu S Chen J Yuan Z Liang P Cui H. Mechanism of notch signaling pathway in malignant progression of glioblastoma and targeted therapy. Biomolecules. (2024) 14:480. doi: 10.3390/biom14040480, PMID: 38672496 PMC11048644

[31] Liu C Ge H Shen C Hu D Zhao X Qin R. NOTCH3 promotes malignant progression of bladder cancer by directly regulating SPP1 and activating PI3K/AKT pathway. Cell Death Dis. (2024) 15:840. doi: 10.1038/s41419-024-07241-0, PMID: 39557868 PMC11574029

[32] Perlman BS Burget N Zhou Y Schwartz GW Petrovic J Modrusan Z. Enhancer-promoter hubs organize transcriptional networks promoting oncogenesis and drug resistance. Nat Commun. (2024) 15:8070. doi: 10.1038/s41467-024-52375-6, PMID: 39277592 PMC11401928

[33] Reedijk M Odorcic S Chang L Zhang H Miller N McCready DR. High-level Coexpression of JAG1 and NOTCH1 is observed in human breast Cancer and is associated with poor overall survival. Cancer Res. (2005) 65:8530–7. doi: 10.1158/0008-5472.CAN-05-1069, PMID: 16166334

[34] Shen Q Cohen B Zheng W Rahbar R Martin B Murakami K. Notch shapes the innate immunophenotype in breast cancer. Cancer Discov. (2017) 7:1320–35. doi: 10.1158/2159-8290.CD-17-0037, PMID: 28790030

[35] Bu P Wang L Chen K-Y Srinivasan T Kadur P Murthy L. A miR-34a-numb feedforward loop triggered by inflammation regulates asymmetric stem cell division in intestine and colon cancer. Cell Stem Cell. (2016) 18:189–202. doi: 10.1016/j.stem.2016.01.006, PMID: 26849305 PMC4751059

[36] Gonulcu SC Unal B Bassorgun IC Ozcan M Coskun HS Elpek GO. Expression of notch pathway components (numb, itch, and Siah-1) in colorectal tumors: a clinicopathological study. World J Gastroenterol. (2020) 26:3814–33. doi: 10.3748/wjg.v26.i26.3814, PMID: 32774060 PMC7383841

[37] Ruland J. Colon Cancer: epithelial notch signaling recruits neutrophils to drive metastasis. Cancer Cell. (2019) 36:213–4. doi: 10.1016/j.ccell.2019.08.010, PMID: 31526756

[38] Stephens PJ Davies HR Mitani Y Van Loo P Shlien A Tarpey PS. Whole exome sequencing of adenoid cystic carcinoma. J Clin Invest. (2013) 123:2965–8. doi: 10.1172/JCI67201, PMID: 23778141 PMC3999050

[39] Xie M Wei S Wu X Li X You Y He C. Alterations of notch pathway in patients with adenoid cystic carcinoma of the trachea and its impact on survival. Lung Cancer. (2018) 121:41–7. doi: 10.1016/j.lungcan.2018.04.020, PMID: 29858025

[40] Boelens MC Wu TJ Nabet BY Xu B Qiu Y Yoon T. Exosome transfer from stromal to breast cancer cells regulates therapy resistance pathways. Cell. (2014) 159:499–513. doi: 10.1016/j.cell.2014.09.051, PMID: 25417103 PMC4283810

[41] Lu Z Ren Y Zhang M Fan T Wang Y Zhao Q. FLI-06 suppresses proliferation, induces apoptosis and cell cycle arrest by targeting LSD1 and notch pathway in esophageal squamous cell carcinoma cells. Biomed Pharmacother. (2018) 107:1370–6. doi: 10.1016/j.biopha.2018.08.140, PMID: 30257352

[42] Yang H Xiang Y Tan T Lei Y. ORY-1001 inhibits glioblastoma cell growth by downregulating the notch/HES1 pathway via suppressing lysine-specific demethylase 1 expression. J South Med Univ. (2024) 44:1620–30. doi: 10.12122/j.issn.1673-4254.2024.08.22, PMID: 39276059 PMC11378054

[43] Ali SA Justilien V Jamieson L Murray NR Fields AP. Protein kinase Cι drives a NOTCH3-dependent stem-like phenotype in mutant KRAS lung adenocarcinoma. Cancer Cell. (2016) 29:367–78. doi: 10.1016/j.ccell.2016.02.012, PMID: 26977885 PMC4795153

[44] Fan X Khaki L Zhu TS Soules ME Talsma CE Gul N. Notch pathway blockade depletes CD133-positive glioblastoma cells and inhibits growth of tumor neurospheres and xenografts. Stem Cells. (2009) 28:5–16. doi: 10.1002/stem.254, PMID: 19904829 PMC2878196

[45] Liu H Wang J Zhang M Xuan Q Wang Z Lian X. Jagged1 promotes aromatase inhibitor resistance by modulating tumor-associated macrophage differentiation in breast cancer patients. Breast Cancer Res Treat. (2017) 166:95–107. doi: 10.1007/s10549-017-4394-2, PMID: 28730338

[46] Zhang N Yin R Zhou P Liu X Fan P Qian L. DLL1 orchestrates CD8 T cells to induce long-term vascular normalization and tumor regression. Proc Natl Acad Sci U S A. (2021) 118:e2020057118. doi: 10.1073/pnas.2020057118, PMID: 34035167 PMC8179177

[47] Geng Y Fan J Chen L Zhang C Qu C Qian L. A notch-dependent inflammatory feedback circuit between macrophages and Cancer cells regulates pancreatic Cancer metastasis. Cancer Res. (2021) 81:64–76. doi: 10.1158/0008-5472.CAN-20-0256, PMID: 33172931

[48] Yang M Zhang G Wang Y He M Xu Q Lu J. Tumour-associated neutrophils orchestrate intratumoural IL-8-driven immune evasion through Jagged2 activation in ovarian cancer. Br J Cancer. (2020) 123:1404–16. doi: 10.1038/s41416-020-1026-0, PMID: 32778818 PMC7591527

[49] Mao L Zhao Z-L Yu G-T Wu L Deng W-W Li Y-C. Γ-Secretase inhibitor reduces immunosuppressive cells and enhances tumour immunity in head and neck squamous cell carcinoma. Int J Cancer. (2018) 142:999–1009. doi: 10.1002/ijc.31115, PMID: 29047105

[50] Zeng J Zhang Y Fang Y Lian J Zhang H Zhang S. Natural product Quercetin-3-methyl ether promotes colorectal cancer cell apoptosis by downregulating intracellular polyamine signaling. Int J Med Sci. (2024) 21:904–13. doi: 10.7150/ijms.93903, PMID: 38617002 PMC11008483

[51] Chen X Dong X-S Gao H-Y Jiang Y-F Jin Y-L Chang Y-Y. Suppression of HSP27 increases the anti-tumor effects of quercetin in human leukemia U937 cells. Mol Med Rep. (2016) 13:689–96. doi: 10.3892/mmr.2015.4600, PMID: 26648539 PMC4686121

[52] Li Y Wang Z Jin J Zhu S-X He G-Q Li S-H. Quercetin pretreatment enhances the radiosensitivity of colon cancer cells by targeting Notch-1 pathway. Biochem Biophys Res Commun. (2020) 523:947–53. doi: 10.1016/j.bbrc.2020.01.048, PMID: 31964531

[53] Ahmadi L Eskandari N Ghanadian M Rahmati M Kasiri N Etamadifar M. The immunomodulatory aspect of quercetin penta acetate on Th17 cells proliferation and gene expression in multiple sclerosis. Cell J (Yakhteh). (2023) 25:110–7. doi: 10.22074/cellj.2022.557560.1073, PMID: 36840457 PMC9968368

[54] Cheng W Zhang H Xiao L. Clinical study of Semem litchi in the treatment of breast hyperplasia. Clin Med Eng. (2013) 20:25–6. doi: 10.3969/j.issn.1674-4659.2013.01.0025

[55] Wang Z Zhou J Liu S Wan D Pei-wen LI. Li Pei-wen’s experience on the treatment of breast cancer with triangular medicine. China J Tradit Chin Med Pharm. (2022) 37:2065–8.

[56] Liao Y Luo Z Liu Y Xue W He S Chen X. Total flavonoids of Litchi seed attenuate stem cell-like properties in breast cancer by regulating Notch3 signaling pathway. J Ethnopharmacol. (2023) 305:116133. doi: 10.1016/j.jep.2023.116133, PMID: 36603788

[57] Zhu W Zhu Y Xu H Wang T Wang J Meng M. Flavone inhibited proliferation of T-ALL by promoting c-Cbl-induced ubiquitinylation and degradation of Notch1. Biochem Biophys Res Commun. (2020) 522:684–9. doi: 10.1016/j.bbrc.2019.11.148, PMID: 31785807

[58] Patel NK Bhutani KK. Pinostrobin and *Cajanus cajan* (L.) leaves inhibits TNF-α and IL-1β production: in vitro and in vivo experimentation. Phytomedicine. (2014) 21:946–53. doi: 10.1016/j.phymed.2014.02.011, PMID: 24680612

[59] Tiwari RK Ahmad A Khan MS Shahanawaz SD Ahmad S Ansari IA. Pinostrobin suppresses the proliferation of lung carcinoma cells by abrogating the cell cycle progression through the inhibition of notch signaling pathway. S Afr J Bot. (2022) 151:614–22. doi: 10.1016/j.sajb.2022.08.030

[60] Hardeep ST Vaishali A Gaurav P Diwakar A Nidarshana CP Muobarak JT. Xanthohumol: a metabolite with promising anti-neoplastic potential. Anti Cancer Agents Med Chem. (2022) 22:418–32. doi: 10.2174/1871520621666210223095021, PMID: 33622230

[61] Kunnimalaiyaan S Trevino J Tsai S Gamblin TC Kunnimalaiyaan M. Xanthohumol-mediated suppression of Notch1 signaling is associated with antitumor activity in human pancreatic Cancer cells. Mol Cancer Ther. (2015) 14:1395–403. doi: 10.1158/1535-7163.MCT-14-0915, PMID: 25887885 PMC4554525

[62] Hossain MM Banik NL Ray SK. Survivin knockdown increased anti-cancer effects of (−)-epigallocatechin-3-gallate in human malignant neuroblastoma SK-N-BE2 and SH-SY5Y cells. Exp Cell Res. (2012) 318:1597–610. doi: 10.1016/j.yexcr.2012.03.033, PMID: 22507272 PMC3374045

[63] Jin H Gong W Zhang C Wang S. Epigallocatechin gallate inhibits the proliferation of colorectal cancer cells by regulating notch signaling. Onco Targets Ther. (2013) 6:145–53. doi: 10.2147/OTT.S40914, PMID: 23525843 PMC3596123

[64] Zhu Q-Q Yang X-Y Zhang X-J Yu C-J Pang Q-Q Huang Y-w. EGCG targeting notch to attenuate renal fibrosis via inhibition of TGFβ/Smad3 signaling pathway activation in streptozotocin-induced diabetic mice. Food Funct. (2020) 11:9686–95. doi: 10.1039/d0fo01542c, PMID: 33057539

[65] Wei H Ge Q Zhang L-Y Xie J Gan R-H Lu Y-G. EGCG inhibits growth of tumoral lesions on lip and tongue of K-Ras transgenic mice through the notch pathway. J Nutr Biochem. (2022) 99:108843. doi: 10.1016/j.jnutbio.2021.108843, PMID: 34407449

[66] Li Q Qu B Shen H Deng H Sun L. Histone demethylase GASC1 inhibitor targeted GASC1 gene to inhibit the malignant transformation of esophageal cancer through the NOTCH-MAPK signaling pathway. Ann Clin Lab Sci. (2022) 52:240–8. PMID: 35414503

[67] Giordano F D’Amico M Montalto FI Malivindi R Chimento A Conforti FL. Cdk4 regulates glioblastoma cell invasion and Stemness and is target of a notch inhibitor plus resveratrol combined treatment. Int J Mol Sci. (2023) 24:10094. doi: 10.3390/ijms241210094, PMID: 37373242 PMC10298906

[68] Patra S Pradhan B Nayak R Behera C Das S Patra SK. Dietary polyphenols in chemoprevention and synergistic effect in cancer: clinical evidences and molecular mechanisms of action. Phytomedicine. (2021) 90:153554. doi: 10.1016/j.phymed.2021.153554, PMID: 34371479

[69] Giordano F Montalto FI Panno ML Andò S De Amicis F. A notch inhibitor plus resveratrol induced blockade of autophagy drives glioblastoma cell death by promoting a switch to apoptosis. Am J Cancer Res. (2021) 11:5933. PMID: 35018234 PMC8727809

[70] Cai J Qiao Y Chen L Lu Y Zheng D. Regulation of the notch signaling pathway by natural products for cancer therapy. J Nutr Biochem. (2024) 123:109483. doi: 10.1016/j.jnutbio.2023.109483, PMID: 37848105

[71] Yoganathan S Alagaratnam A Acharekar N Kong J. Ellagic acid and Schisandrins: natural Biaryl polyphenols with therapeutic potential to overcome multidrug resistance in Cancer. Cells. (2021) 10:458. doi: 10.3390/cells10020458, PMID: 33669953 PMC7924821

[72] Gupta A Singh AK Kumar R Jamieson S Pandey AK Bishayee A. Neuroprotective potential of Ellagic acid: a critical review. Adv Nutr. (2021) 12:1211–38. doi: 10.1093/advances/nmab007, PMID: 33693510 PMC8321875

[73] Wang D Chen Q Tan Y Liu B Liu C. Ellagic acid inhibits human glioblastoma growth in vitro and in vivo. Oncol Rep. (2017) 37:1084–92. doi: 10.3892/or.2016.5331, PMID: 28035411

[74] Peiffer DS Ma E Wyatt D Albain KS Osipo C. DAXX-inducing phytoestrogens inhibit ER+ tumor initiating cells and delay tumor development. NPJ Breast Cancer. (2020) 6:37. doi: 10.1038/s41523-020-00178-5, PMID: 32864429 PMC7429502

[75] Gupta AP Khan S Manzoor MM Yadav AK Sharma G Anand R. Anticancer curcumin: natural analogues and structure-activity relationship In: Attaur R, editor. Studies in natural products chemistry. Amsterdam, Netherlands: Elsevier (2017). 355–401. doi: 10.1016/B978-0-444-63929-5.00010-3

[76] Chen J Xu T Chen C. The critical roles of miR-21 in anti-cancer effects of curcumin. Ann Transl Med. (2015) 3:330. doi: 10.3978/j.issn.2305-5839.2015.09.20, PMID: 26734640 PMC4691004

[77] Liao S Xia J Chen Z Zhang S Ahmad A Miele L. Inhibitory effect of curcumin on oral carcinoma CAL-27 cells via suppression of Notch-1 and NF-κB signaling pathways. J Cell Biochem. (2011) 112:1055–65. doi: 10.1002/jcb.23019, PMID: 21308734

[78] Liu Z-C Yang Z-X Zhou J-S Zhang H-T Huang Q-K Dang L-L. Curcumin regulates hepatoma cell proliferation and apoptosis through the notch signaling pathway. Int J Clin Exp Med. (2014) 7:714. PMID: 24753768 PMC3992413

[79] Liubomirski Y Ben-Baruch A. Notch-inflammation networks in regulation of breast Cancer progression. Cells. (2020) 9:1576. doi: 10.3390/cells9071576, PMID: 32605277 PMC7407628

[80] McCaw TR Inga E Chen H Jaskula-Sztul R Dudeja V Bibb JA. Gamma secretase inhibitors in Cancer: a current perspective on clinical performance. Oncologist. (2021) 26:e608–21. doi: 10.1002/onco.13627, PMID: 33284507 PMC8018325

[81] Song C Zhang J Xu C Gao M Li N Geng Q. The critical role of γ-secretase and its inhibitors in cancer and cancer therapeutics. Int J Biol Sci. (2023) 19:5089–103. doi: 10.7150/ijbs.87334, PMID: 37928268 PMC10620818

[82] Fahad K Vipendra KS Mohd S Mohd AK Irfan AA. Carvacrol induced program cell death and cell cycle arrest in androgen-independent human prostate cancer cells via inhibition of notch signaling. Anti Cancer Agents Med Chem. (2019) 19:1588–608. doi: 10.2174/1871520619666190731152942, PMID: 31364516

[83] Malla R Marni R Chakraborty A Kamal MA. Diallyl disulfide and diallyl trisulfide in garlic as novel therapeutic agents to overcome drug resistance in breast cancer. J Pharm Anal. (2022) 12:221–31. doi: 10.1016/j.jpha.2021.11.004, PMID: 35582397 PMC9091922

[84] Liu X Liang Q Wang Y Xiong S Yue R. Advances in the pharmacological mechanisms of berberine in the treatment of fibrosis. Front Pharmacol. (2024) 15:5058. doi: 10.3389/fphar.2024.1455058, PMID: 39372209 PMC11450235

[85] Palaga T Wongchana W Kueanjinda P. Notch signaling in macrophages in the context of Cancer immunity. Front Immunol. (2018) 9:652. doi: 10.3389/fimmu.2018.00652, PMID: 29686671 PMC5900058

[86] Yang P Zhou X Xie Y. Cytotoxic effects of the Benzophenanthridine alkaloids isolated from *Eomecon chionantha* on MCF-7 cells and its potential mechanism. Chem Biodivers. (2023) 20:e202200871. doi: 10.1002/cbdv.202200871, PMID: 36529680

