# Supplementary figures and images for: Interaction between Notch signaling pathway and bioactive compounds and its intervention on cancer
Source: Front Nutr. 2025 Sep 23;12:1647661. doi: 10.3389/fnut.2025.1647661 (PMC12500663; doi:10.3389/fnut.2025.1647661)

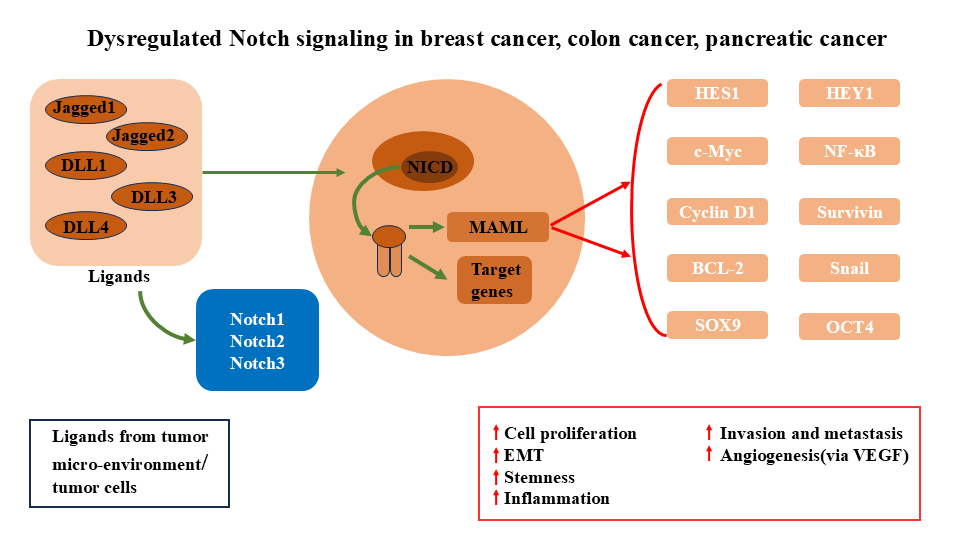

Supplement: Supplementary file 1 [file Image_1.tif]
